# Lowest instrumented vertebrae in early onset scoliosis: is there a role for a more selective approach?

**DOI:** 10.1007/s43390-024-00842-x

**Published:** 2024-03-21

**Authors:** Michael J. Heffernan, Claudia Leonardi, Lindsay M. Andras, Bailli Fontenot, Luke Drake, Joshua M. Pahys, John T. Smith, Peter F. Sturm, George H. Thompson, Michael P. Glotzbecker, Tyler A. Tetreault, Benjamin D. Roye, Pediatric Spine Study Group, Ying Li

**Affiliations:** 1 Jackie and Gene Autry Orthopaedic Center, Children’s Hospital Los Angeles, 4650 Sunset Blvd, Mailstop #69, Los Angeles, CA 90027 USA; 2 School of Public Health, LSU Health Sciences Center, New Orleans, LA USA; 3 LSU Health Sciences Center, Children’s Hospital New Orleans, New Orleans, LA USA; 4 Department of Orthopedics, Shriners Hospital for Children, Philadelphia, PA USA; 5 Department of Orthopaedics, University of Utah, Salt Lake City, UT USA; 6 Cincinnati Children’s Hospital Medical Center, University of Cincinnati, Cincinnati, OH USA; 7 Rainbow Babies and Children’s Hospital, Case Western Reserve University, Cleveland, OH USA; 8 Department of Orthopaedic Surgery, Columbia University Medical Center, New York, NY USA; 9 Pediatric Orthopaedic Surgery, New York-Presbyterian Morgan Stanley Children’s Hospital, New York, NY USA; 10 Department of Orthopaedic Surgery, C.S. Mott Children’s Hospital, Ann Arbor, MI USA

**Keywords:** Lowest instrumented vertebrae, Early-onset scoliosis, LIV selection, Growth-friendly instrumentation

## Abstract

**Purpose:**

This purpose of this study was to assess the impact of patient and implant characteristics on LIV selection in ambulatory children with EOS and to assess the relationship between the touched vertebrae (TV), the last substantially touched vertebrae (LSTV), the stable vertebrae (SV), the sagittal stable vertebrae (SSV), and the LIV.

**Methods:**

A multicenter pediatric spine database was queried for patients ages 2–10 years treated by growth friendly instrumentation with at least 2-year follow up. The relationship between the LIV and preoperative spinal height, curve magnitude, and implant type were assessed. The relationships between the TV, LSTV, SV, SSV, and the LIV were also evaluated.

**Results:**

Overall, 281 patients met inclusion criteria. The LIV was at L3 or below in most patients with a lumbar LIV: L1 (9.2%), L2 (20.2%), L3 (40.9%), L4 (29.5%). Smaller T1 − T12 length was associated with more caudal LIV selection (*p* = 0.001). Larger curve magnitudes were similarly associated with more caudal LIV selection (*p* = < 0.0001). Implant type was not associated with LIV selection (*p* = 0.32) including MCGR actuator length (*p* = 0.829). The LIV was caudal to the TV in 78% of patients with a TV at L2 or above compared to only 17% of patients with a TV at L3 or below (*p* < 0.0001).

**Conclusions:**

Most EOS patients have an LIV of L3 or below and display TV–LIV and LSTV–LIV incongruence. These findings suggest that at the end of treatment, EOS patients rarely have the potential for selective thoracic fusion. Further work is necessary to assess the potential for a more selective approach to LIV selection in EOS.

**Level of evidence:**

III.

## Introduction

Selection of the lowest instrumented vertebrae (LIV) is a foundational principle guiding the management of spinal deformity [1]. In adolescent idiopathic scoliosis (AIS), classification systems exist to assist with LIV selection to limit the extension of spinal fusion while achieving excellent spinal correction and balance [1–4]. There has been less emphasis on LIV selection in early-onset scoliosis (EOS) treated with distraction-based growth-friendly instrumentation (GFI) [5, 6]. The variability of curve patterns and diagnoses found within this population may complicate the process of LIV selection [7]. It is also possible that the size and space requirements of the implants used in GFI limit LIV selection options [6].

There are a host of studies surrounding LIV selection in AIS [1, 8, 9]. Key factors influencing LIV decisions include the last touched vertebrae (TV), curve flexibility on pre-operative bending films, rotation at the apex, curve magnitude, and clinical exam [1, 2, 8, 9]. In contrast, there is a paucity of literature evaluating LIV selection in EOS, and little is known about the factors influencing LIV decisions in this patient population [5–7].

The purpose of this study was to assess the impact of patient, implant, and curve characteristics on LIV selection in ambulatory children with EOS. We hypothesized that implant type and patient size would inform LIV selection. Due to the rigid portion of the actuator, we anticipated that magnetically controlled growing rods (MCGR) would be associated with a more caudal LIV when compared to traditional growing rods (TGR) and vertical expandable prosthetic titanium rib (VEPTR). We also hypothesized that a substantial mismatch would exist between LIV selection and both the TV and the last substantially touched vertebrae (LSTV) on preoperative radiographs with longer constructs being frequently encountered.

## Methods

This was a retrospective review of an institutional review board-approved multicenter international database of patients diagnosed with early onset scoliosis and treated with growth friendly instrumentation.

### Eligibility and selection criteria

**Fig. 1:**
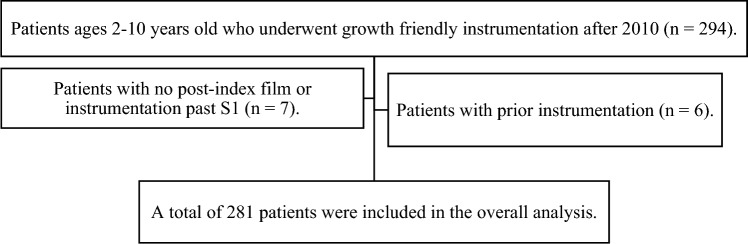
Exclusion flow chart

Ambulatory EOS patients with an idiopathic diagnosis between the ages of 2 and 10 years treated with TGR, VEPTR, and MCGR between 2010 and 2020 with minimum 2 year follow up were screened for eligibility. Patients with incomplete data, instrumentation extending to S1 or below, or listed as having had prior surgical instrumentation were excluded from the study (Fig. 1).

### Data collection

Baseline patient characteristics collected included age, sex, race, weight, and height. Pre-index data included major and minor curve magnitudes and thoracic spine heights (TSH) from T1–T12 and total spine height from T1–S1. Operative details, including date of surgery, instrumentation type (MCGR vs. VEPTR/TGR), and MCGR actuator size (70 vs. 90 mm) were recorded. A center sacral vertical line (CSVL) was drawn on upright preoperative anterior–posterior radiographs to assess the last touched vertebrae (TV), the last substantially touched vertebrae (LSTV), and the stable vertebrae (SV). The TV was defined as the most cephalad thoracolumbar or lumbar vertebra that was “touched” by the CSVL on any portion of the involved vertebrae. The LSTV was defined as the most cephalad thoracolumbar or lumbar vertebrae that the CSVL at least touched or was medial to the lateral border of the pedicle. The SV was defined as the most cephalad vertebrae below the curve apex that was most closely bisected by the CSVL. The posterior sacral vertical line was drawn on upright preoperative lateral radiographs to assess the stable sagittal vertebrae (SSV), which was defined as the most cephalad vertebrae at which 50% of the vertebral body was in front of the posterior sacral vertical line. The primary outcome was LIV, which was defined as the most caudal instrumented vertebrae as determined by postoperative radiographs. Secondary outcomes included factors associated with the LIV as well as the relationship between the TV, LSTV, SV, SSV and the LIV.

### Study design

Due to the lack of a previous multi-center study of LIV selection in EOS, we first established the relative LIV distribution in ambulatory patients with EOS. We then assessed the LIV in relation to both implant and patient factors including preoperative spinal height, curve magnitude, implant type, and actuator size. Finally, we analyzed the relationship between TV, LSTV, SV, SSV, and the LIV.

### Data analysis

Data were analyzed using SAS/STAT software version 9.4 (SAS Institute Inc., Cary, NC). The association between LIV and selected patient demographics and clinical characteristics within lumbar vertebrae was compared using ANOVA and Tukey adjustment for multiple comparisons when an overall significant association was observed. The LIV was compared between surgical instrumentation type (MCGR vs. VEPTR/TGR) and MCGR actuator length (70 vs. 90 mm) using the *χ*^2^ test for categorical variables. Where applicable, residuals were independent with an identical and normal distribution and homogenous variances. A 2-sided *p* < 0.05 indicated statistical significance.

## Results

Two hundred eighty-one patients met the inclusion criteria, of which 165 were treated with MCGR and 116 were treated with VEPTR/TGR. Demographics and pre-index radiographic measures are shown in Table 1. Of note, 65% of patients underwent surgery in more recent years (between 2015 and 2020).

**Table 1:** Patient demographics and pre index surgery clinical characteristics

Characteristics	*n*	All patients Mean (SD)
Age, years	281	6.7 (2.2)
Weight, kg	251	22.8 (9.4)
Height, cm	253	116.4 (17.2)
Major Cobb angle, °	268	73.7 (20.8)
Minor Cobb angle, °	252	44.1 (12.9)
Thoracic spine height (T1 − T12), cm	229	16.7 (3.2)
Thoracic spine height (T1 − S1), cm	228	27.1 (4.7)
Kyphosis, °	237	49.3 (21.1)

	*n*	%
Sex		
Female	177	63.0
Male	104	37.0
Race		
Black/African American	43	16.3
White/Caucasian	178	67.7
Other	42	16.0
Surgery year		
2010–2015	98	34.9
2015–2020	183	65.1

*SD* standard deviation

**Fig. 2:**
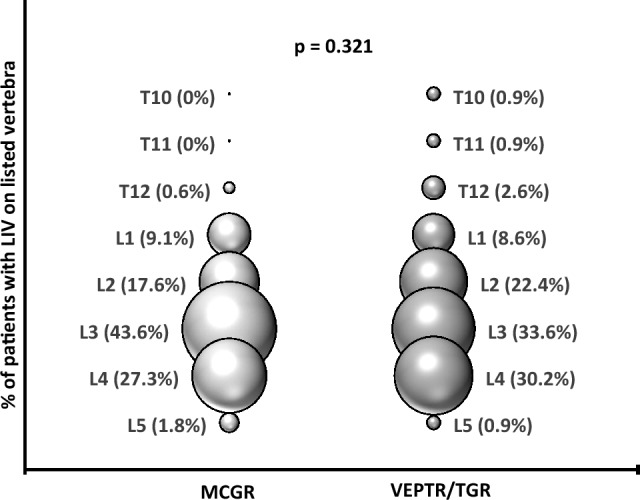
Percentage of patients (bubble width) with lowest instrumented vertebrae (LIV) on listed vertebra by index surgery type [magnetically controlled growing rods (MCGR) vs. vertical expandable prosthetic titanium rib or traditional growing rods (VEPTR/TGR)]

Two hundred seventy-one patients (96.4%) had a lumbar LIV between L1 and L4. The LIV was most often at the L3 vertebra, encompassing 40.9% of lumbar LIVs. Other lumbar LIV levels included L4 (29.5%), L2 (20.2%), L1 (9.2%) in decreasing order. Thoracic level 10, T11, T12 and lumbar level L5 accounted for a small number of reported LIV selections (3.5%). LIV selection was not associated with implant type (MCGR vs. TGR/VEPTR, *p* = 0.321) (Fig. 2) or actuator length (70 vs. 90 mm, *p* = 0.829) within MCGR.

The association between LIV and selected patient characteristics (Table 2) was investigated in patients with LIV of L1–L4 as instrumentation ending in the thoracic spine or distally at L5 was observed in very few patients (*n* = 10). LIV selection was associated with pre-index major and minor curve magnitudes (*p* < 0.001) and spinal height as measured by T1 − T12 spinal length (*p* = 0.001), T1 − S1 spinal length (*p* = 0.001), and kyphosis (*p* < 0.001). Neither patient age (*p* = 0.569) or clinically measured height (*p* = 0.188) were significantly associated with LIV selection. As the LIV level extended caudally, pre-index major and minor curve magnitudes as well as kyphosis increased while thoracic spinal height decreased almost linearly, indicating that a more caudal LIV was chosen in patients with smaller trunk heights and those with more pronounced spinal deformity.

**Table 2:** Patient demographics and pre index surgery clinical characteristics within lumbar vertebrae by LIV

Characteristics	L1 (*n* = 25)	L2 (*n* = 55)	L3 (*n* = 111)	L4 (*n* = 80)	*p* value
Age, years	7.3 (2.2)	6.7 (2.4)	6.6 (2.1)	6.6 (2.3)	0.569
Height, cm (*n* = 245)	120.8 (16.9)	119.0 (17.8)	114.2 (15.9)	114.9 (18.1)	0.188
Major Cobb angle, ° (*n* = 259)	61.0^c^ (11.1)	67.4^bc^ (18.0)	74.8^ab^ (20.7)	81.8^a^ (21.9)	< 0.0001
Minor Cobb angle, ° (*n* = 243)	37.8^b^ (10.0)	39.2^b^ (11.7)	44.1^b^ (11.9)	49.3^a^ (13.7)	< 0.0001
TSH (T1–T12), cm (*n* = 221)	18.5^a^ (3.4)	17.5^ab^ (3.6)	16.2^b^ (2.8)	16.0^b^ (3.2)	0.001
TSH (T1–S1), cm (*n* = 220)	30.2^a^ (5.2)	28.1^ab^ (4.7)	26.4^b^ (4.0)	25.9^b^ (4.7)	0.001
Kyphosis, ° (*n* = 229)	37.9b (4.3)	42.5b (3.0)	49.8b (2.1)	58.2a (2.5)	< 0.0001

*SD* standard deviation, *TSH* total spine height

**Fig. 3:**
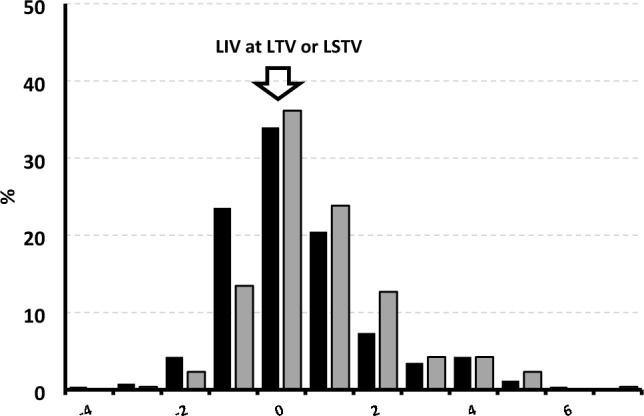
Percentage of patients with lowest instrumented vertebrae (LIV) at last touched vertebra (LTV, gray) or at last substantially touched vertebra (LSTV, black), caudal (+ 1, 2, 3, 4, 5, 6, or 7) and proximal (− 4, − 3, − 2, or − 1)

The majority (63.8%) of patients exhibited some degree of LIV-TV level incongruence (Fig. 3) and 47.6% of patients had an LIV below the TV (+ 1, + 2, …, and + 7). In most patients (64.5%), the TV and LSTV were the same vertebral level. As such, we found similar trends in LIV-LSTV incongruency and 37% had an LIV that was caudal to the LSTV (Fig. 3).

**Fig. 4:**
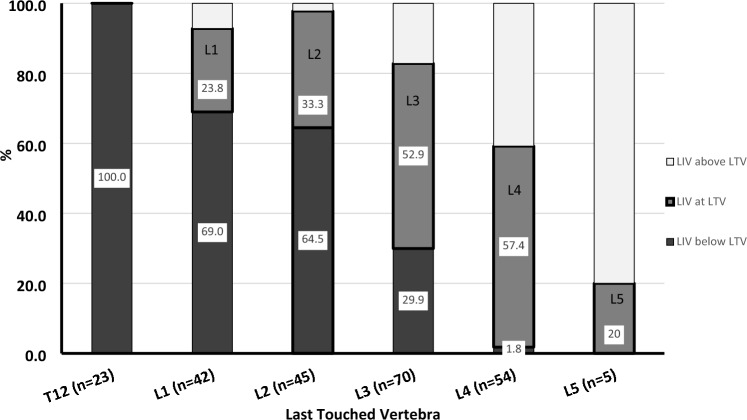
Percentage of patients with lowest instrumented vertebrae (LIV) above (white), at (gray) and below (black) the last touched vertebra (LTV) presented by LTV. Analysis limited to LTV from T12 to L5

**Fig. 5:**
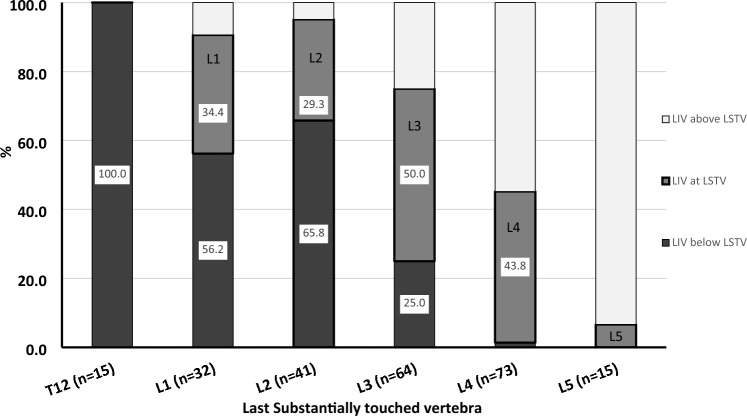
Percentage of patients with lowest instrumented vertebrae (LIV) above (white), at (gray) and below (black) the last substantially touched vertebra (LSTV) presented by LSTV

**Fig. 6:**
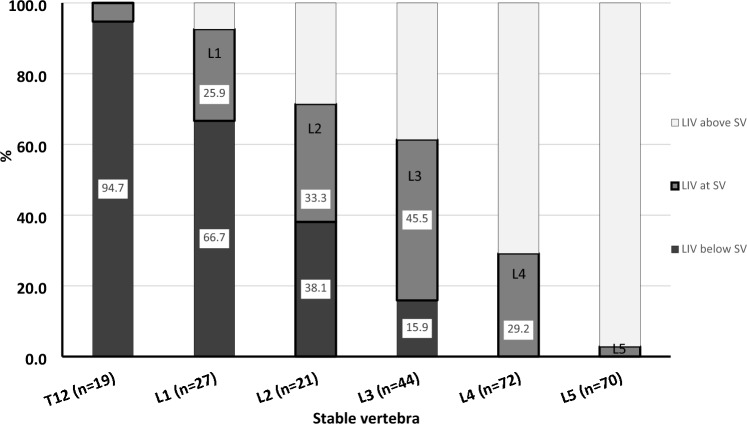
Percentage of patients with lowest instrumented vertebrae (LIV) above (white), at (gray) and below (black) the stable vertebra (SV) presented by SV

**Fig. 7:**
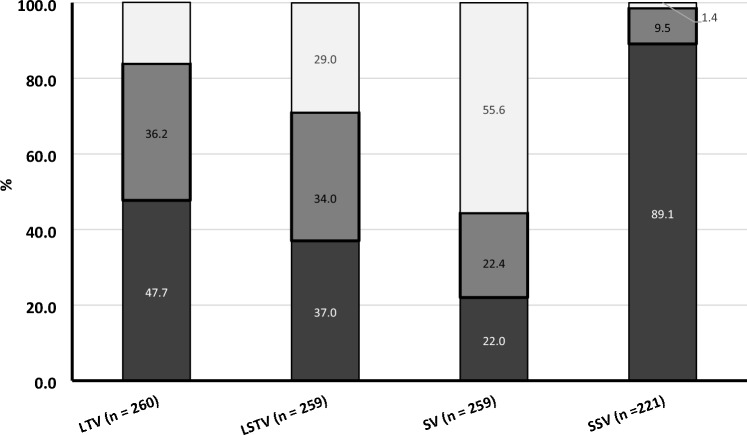
Percentage of patients with lowest instrumented vertebrae (LIV) above (white), at (gray) and below (black) the last touched vertebra (LTV), the last substantially touched vertebra (LSTV), stable vertebra (SV), and sagittal stable vertebrae (SSV)

We further explored the association between the LIV and both the TV (Fig. 4) and LSTV (Fig. 5). As the TV was located more cranial (T12 and L1), the incidence of LIV-TV incongruence increased with the LIV invariably being caudal to the TV. LIV–TV congruence increased with lower TV levels (bolded box). LIV–TV congruence peaked at L4, where nearly 58% of the patients had their LIV at the same level as the TV. The LIV was caudal to the TV in 78% of patients with a TV at L2 or above compared to only 17% of patients with a TV at L3 or below (*p* < 0.0001). A similar trend was seen when comparing the LSTV and LIV (Fig. 5), although the vertebrae of maximal congruence was L3 when analyzing by LSTV. When assessing by SV (Fig. 6), the LIV was below the SV if the SV was at L2 (38%) or above (> 67%). Due to the overall tendency to select the LIV at either L3 or L4, when the TV or LSTV occurred at a more cranial vertebra, the LIV tended to be more caudal than either the TV or the LSTV. The SSV tended to be more cephalad when compared to the coronal parameters (Table 3). The vast majority (89%) of LIV selections were below the SSV (Fig. 7).

**Table 3:** Distribution of coronal and sagittal parameters

Vertebra	LTV	LSTV	SV	SSV	Apex of kyphosis
T3	–	–	–	0.5 (1)	1.4 (3)
T4	–	–	–	0.5 (1)	3.2 (7)
T5	–	–	–	8.6 (19)	10.4 (23)
T6	–	–	–	0.5 (1)	18.1 (40)
T7	–	–	–	4.1 (9)	19.0 (42)
T8	–	–	–	4.1 (9)	20.4 (45)
T9	0.4 (1)	–	–	5.0 (11)	17.2 (38)
T10	3.1 (8)	1.9 (5)	0.8 (2)	9.5 (21)	5.0 (11)
T11	4.6 (12)	5.4 (14)	1.5 (4)	14.9 (33)	3.2 (7)
T12	8.9 (23)	5.8 (15)	7.3 (19)	14.0 (31)	1.4 (3)
L1	16.2 (42)	12.4 (32)	10.4 (27)	14.0 (31)	0.9 (2)
L2	17.3 (45)	15.8 (41)	8.1 (21)	14.9 (33)	–
L3	26.9 (70)	24.7 (64)	17.0 (44)	7.2 (16)	–
L4	20.8 (54)	28.2 (73)	27.8 (72)	2.3 (5)	–
L5	1.9 (5)	5.8 (15)	27.0 (70)	–	–

*LTV* last touched vertebrae, *LSTV* last substantially touched vertebrae, *SV* stable vertebrae, *SSV* sagittal stable vertebrae

The LIV after definitive fusion was available for 82 patients (29%). Of those patients with documented definitive fusion after initial growth friendly instrumentation, there was no change in the LIV for most patients with an initial LIV of L3 (no change in 82%) or L4 (92%). In contrast, a more cephalad index LIV was associated with a caudal shift in LIV following definitive fusion. The percent of patients with a more caudal LIV after definitive fusion was: L1 (67%), L2 (38%), L3 (18%), L4 (8%). Only one patient, whose index LIV was at L4, had extension to the pelvis at most recent follow-up.

## Discussion

This study represents the largest multicenter analysis of the lowest instrumented vertebrae in early onset scoliosis. Although selective thoracic surgery is an area of focus in adolescent idiopathic scoliosis, these results show that it is a rare occurrence in EOS. In this cohort, only 11% of patients had a LIV at L1 or cephalad. The most common LIV was L3 and the majority of patients had an LIV of L3 or L4. We attempted to identify the underlying reasons for LIV selection and hypothesized that patient size and implant type would obligate a lower LIV. Larger curve magnitude and shorter spinal height were associated with a more caudal LIV, however, there was no association between implant type and LIV. We also found a significant incongruence between the TV, LSTV, and the LIV. Only 34% of patients had LIV-LSTV congruency. The SSV tended to be more cephalad when compared to coronal parameters and did not appear to play a major role in LIV selection in this series. In this generalizable cohort, 48% of patients had an LIV that was caudal to the TV and 37% had an LIV that was caudal to the LSTV which may represent an opportunity to impact surgical planning.

LIV selection is a common topic of discussion and area of research in the treatment of AIS [1, 2, 4, 8, 10–18]. In contrast, there is a relative paucity of literature assessing LIV selection in EOS with research efforts instead focused on implant type, complications, and spinal growth [5, 7, 19–21]. Upper implant level selection and technique has also received deserved attention as implant failure is more frequent at the upper foundation [22]. Fortunately, there are fewer complications associated with the LIV which may explain the lack of prior research. However, LIV selection has long term implications with several studies citing disc degeneration after posterior spinal fusion into the lower lumbar spine [23–25]. The most common LIV in our cohort was L3, followed by L4, which together represented 71% of patients with a lumbar LIV in this cohort. An additional 20% had an LIV at L2 which left a small proportion of patients who had a selective thoracic surgery. These findings suggest that at the end of treatment, EOS patients rarely have the potential for selective thoracic fusion, an important goal in the treatment of patients with other forms of spinal deformity such as AIS.

We theorized that implant type was a determinant in LIV selection. Specifically, we hypothesized that patients treated with MCGR would have more caudad LIVs when compared to patients treated with TGR due to the size of the MCGR actuator. The results of our study did not support this hypothesis, as there were no significant differences in LIV between implant types including assessment of the 70 mm vs 90 mm MCGR actuators. There were, however, several factors associated with LIV selection identified in this study. Larger curve magnitude was associated with a more caudal LIV. Smaller T1 − T12 and T1 − S1 spinal heights were also associated with lower LIV selection. While the LIV did not differ between implant types, this finding suggests that the need to accommodate the lengthening mechanism of growth friendly instrumentation may have played a role in LIV selection regardless of implant type. Surgeons typically aim to place the lengthening mechanism at the straight thoracolumbar junction which may require a more distal LIV. Further, it makes intrinsic sense that spanning a greater proportion of the spine was necessary to accommodate growth friendly instrumentation in smaller patients.

Given the low rate of selective thoracic surgery in this cohort, it is important to assess if there is an opportunity for surgeons to choose a more cephalad LIV based on existing principles of LIV selection. The last touched vertebra is an entity commonly utilized to guide LIV selection in AIS [18]. Beauchamp et al. found that selecting the “touched vertebrae” in Lenke type 1 and type 2 curves led to reasonable LIV position at a minimum follow-up of 5 years [2]. Dede et al. introduced the concept of the “stable-to-be vertebrae” in an effort to minimize the extent of growth friendly constructs and spare motion segments in idiopathic EOS patients [5]. Supine traction radiographs are necessary to properly identify the “stable-to-be vertebrae” which were not consistently available in the database utilized for the current study. While we cannot specifically report on the “stable to be vertebrae”, it is clear that the “stable to be vertebrae” was not a significant factor in the LIV decisions made in this cohort. In fact, there was notable discrepancy between the TV and the LIV with the vast majority of patients having an LIV caudal to the TV. As surgeons seek to save lumbar motion segments whenever possible, these findings represent a potential area of improvement in the treatment of patients with EOS. In our cohort, there were patients with a caudal LIV after definitive fusion relative to the LIV following the index procedure. Understandably, this was more common when the index LIV was cephalad and uncommon when the LIV was at L3 or L4. Although the risk of adding on is generally higher in skeletally immature patients, the majority of these patients will return to the operating room for either rod lengthening, exchange, or definitive fusion and the LIV can be extended distally at the time of the planned surgery. As such, it may be preferable to avoid selecting too distal of a LIV at the index surgery to maintain the potential to preserve lumbar motion segments.

Despite the important findings of this study, there are several limitations that deserve consideration. It is important to note that this work focused on posterior distraction-based instrumentation and, therefore, does not apply to all forms of EOS treatment. We did not have access to preoperative flexibility imaging for the patients in this cohort. While we identified several factors associated with LIV selection, the criteria utilized by each treating surgeon in making an LIV selection was unknown. This study is further limited by institutional data entry into the registry and the potential for selection bias or institutional variability that accompanies all database studies.

This study represents the largest multicenter assessment of LIV selection in early onset scoliosis. The LIV was at L3 or below in the majority of patients in this cohort. Spinal height and curve magnitude appear to play a role in LIV selection; however, we did not find an association between implant type and LIV. The low rate of selective thoracic surgery and the incongruence between the TV, LSTV, and LIV are important findings of this study. Future work should further assess the reasons for TV–LIV and LSTV–LIV incongruence and whether the same outcomes can be achieved with shorter constructs in selected patients, thus avoiding an obligation to long fusions at the end of treatment for EOS patients.

## Data Availability

Data utilized and study analysis are available upon request.
